# Prognostic Factors of Acute Heart Failure: A Regional Population Registry

**DOI:** 10.3390/jcdd13070310

**Published:** 2026-07-06

**Authors:** Juan Asensio-Nogueira, Miguel Rodriguez-Santamarta, Javier Tobar-Ruíz, Pedro Daniel Perdiguero-Martín, Inés Toranzo-Nieto, Clea González-Maniega, Adrián Lozano-Ibáñez, Lucía Moreno-de-Redrojo Cortes, Manuel Carrasco-Moraleja, Álvaro Margalejo-Franco, Andrea Moreno-González, Luis Eduardo Enríquez-Rodríguez, Sebastián Isaza-Arana, Álvaro Roldán-Sevilla, Williams Enrique Hinojosa-Camargo, Cristina Álvarez-Martínez, Sara Martín-Paniagua, María José Ruiz-Olgado, Jose-Angel Perez-Rivera

**Affiliations:** 1 Department of Cardiology, Complejo Asistencial de Burgos, 09006 Burgos, Spain; 2 Department of Cardiology, Hospital Clínico Universitario de Valladolid, 47003 Valladolid, Spain; 3 Department of Cardiology, Complejo Asistencial Universitario, 24008 León, Spain; 4 Department of Cardiology, Hospital Nuestra Señora de Sonsoles, 05004 Ávila, Spain; 5 Department of Cardiology, Hospital Universitario de Salamanca, 37007 Salamanca, Spain; 6 Department of Statistics, Hospital Clínico Universitario de Valladolid, 47003 Valladolid, Spain; 7 Hospital Virgen de la Concha, 49022 Zamora, Spain; 8 Department of Cardiology, Hospital Santa Bárbara, 42005 Soria, Spain; 9 Department of Cardiology, Hospital de Medina del Campo, 47400 Medina del Campo, Spain; 10 Cardiology Unit, Hospital Santos Reyes, 09006 Aranda de Duero, Spain; 11 Facultad de Ciencias de la Salud, Universidad Isabel I, 09006 Burgos, Spain

**Keywords:** acute heart failure, prognosis, registry, Guideline-directed medical therapy, NT-proBNP, comorbidities

## Abstract

Introduction and objectives: Acute heart failure (AHF) is the leading cardiovascular cause of hospitalization and remains associated with high mortality and rehospitalization rates. Contemporary real-world data from cardiology departments are scarce. We aimed to identify admission characteristics associated with one-year outcomes in patients hospitalized with AHF. Methods: RECYLICA is a prospective, multicentre, regional registry including consecutive patients admitted with AHF to cardiology departments across 10 hospitals over a one-year period. Patients were followed for 12 months. The primary endpoint was the composite of all-cause mortality or heart failure (HF) rehospitalization. Results: A total of 602 patients were included (37.0% women; mean age 72.6 ± 12.0 years), of whom 47.4% had heart failure with reduced left ventricular ejection fraction (HFrEF). During follow-up, 83 patients (13.8%) died and 105 (17.4%) were rehospitalized because of HF. Independent predictors of the primary endpoint were elevated admission N-terminal pro-B-type natriuretic peptide (NT-proBNP), atrial fibrillation (AF), chronic kidney disease (CKD), chronic obstructive pulmonary disease (COPD), previous HF, prior implantable cardioverter-defibrillator (ICD) implantation, and higher left ventricular ejection fraction (LVEF). Among patients with HFrEF, less comprehensive implementation of guideline-directed medical therapy (GDMT) at discharge was associated with significantly worse outcomes. Conclusions: In patients hospitalized with AHF, prognosis is primarily determined by comorbidity burden, admission NT-proBNP levels, previous HF, and LVEF. Among patients with HFrEF, more comprehensive implementation of GDMT at discharge was associated with improved clinical outcomes, supporting early optimization of evidence-based therapy during hospitalization.

## 1. Introduction

Acute heart failure (AHF) is defined by the European Society of Cardiology (ESC) guidelines as the rapid onset or worsening of signs and symptoms resulting from impaired cardiac function that requires urgent medical attention [1]. It commonly presents as an unplanned visit to the emergency department or as an emergency hospital admission. Epidemiological studies estimate that AHF accounts for 1–4% of all hospitalizations, imposing a substantial and increasing healthcare burden due to prolonged hospital stays and a high risk of recurrent admissions [2]. This burden is expected to increase further as populations age in developed countries and the prevalence of cardiometabolic comorbidities rises in developing regions adopting Western lifestyles. Together, these trends have contributed to a steady increase in the prevalence of chronic heart failure (HF) and, consequently, in the number of episodes of acute decompensation.

In our setting, observational studies have consistently reported an in-hospital mortality rate exceeding 6% among patients admitted with AHF [3], while the early post-discharge period remains particularly vulnerable. During this phase, patients are at markedly increased risk of both unplanned readmission and all-cause mortality [4,5,6,7]. Consequently, contemporary management strategies for AHF aim not only to stabilize the acute episode but also to reduce rehospitalizations and improve early post-discharge outcomes.

Despite improvements in acute care, the prognosis after an AHF hospitalization remains poor across the full clinical trajectory. Mortality is substantial during the index admission, and the risk of death and rehospitalization remains elevated after discharge at 30 days, 6 months, and 1 year. This highlights the need for prognostic markers that capture both immediate and longer-term risk.

Contemporary cardiovascular registries have substantially improved our understanding of the demographic characteristics, clinical presentation, and management of patients hospitalized with AHF. However, relatively few have provided comprehensive longitudinal data on prognosis or robust analyses of the clinical factors associated with different disease trajectories [5,6,8].

Although several registries have described the clinical characteristics and outcomes of patients with AHF, contemporary regional real-world data from cardiology departments remain scarce. This gap is clinically relevant because patient case mix, referral pathways, and management strategies in cardiology wards may differ considerably from those in Internal Medicine or Emergency Departments. To address this unmet need, the Registro Castilla y León de Insuficiencia Cardiaca Aguda (RECYLICA) was established to systematically evaluate the multifactorial determinants of one-year outcomes in patients admitted with AHF to cardiology departments throughout the Castilla y León region. By providing contemporary real-world evidence from hospitals with different levels of complexity and organizational structures, this registry aims to generate clinically relevant information that may help optimize the management and risk stratification of this high-risk population.

## 2. Patients and Methods

This prospective, multicentre, open-label cohort study was designed to identify prognostic factors in patients hospitalized with AHF. Consecutive adult patients (≥18 years) admitted with a primary diagnosis of AHF, as defined by the current ESC guidelines [1], were prospectively enrolled. Both de novo AHF and acute decompensation of chronic HF were eligible, provided that HF was the primary reason for hospital admission. The diagnosis was established by the attending cardiologists on the basis of clinical assessment, imaging findings, and biomarker measurements, in accordance with current guideline recommendations.

Patient recruitment was conducted across ten hospitals within the Castilla y León healthcare network, all of which had dedicated cardiology departments. The participating centres represented different levels of complexity, referral patterns, and local clinical protocols, thereby providing a broad representation of contemporary real-world clinical practice across the region and enhancing the external validity of the study findings.

Several exclusion criteria were applied to reduce clinical heterogeneity and ensure a more homogeneous study population. Specifically, we excluded: (1) patients admitted to departments other than Cardiology, such as Internal Medicine or Geriatrics, where patient characteristics, clinical pathways, and management strategies may differ substantially; (2) patients presenting with acute coronary syndromes or severe symptomatic bradyarrhythmias requiring permanent pacemaker implantation within seven days before admission, as these conditions represent distinct clinical entities with different pathophysiological mechanisms, therapeutic approaches, and prognostic trajectories. The aim of the present registry was to characterize patients admitted primarily because of AHF rather than HF secondary to an acute coronary event; (3) patients with advanced non-cardiac comorbidities and an estimated life expectancy of less than six months, owing to their potentially confounding influence on long-term outcomes; and (4) patients who died during the index hospitalization or for whom reliable follow-up data could not be obtained because the study was specifically designed to evaluate one-year post-discharge outcomes. Patient recruitment was conducted between November 2021 and November 2022 to minimize the potential influence of seasonal variation in HF admissions.

At hospital admission, comprehensive baseline data were collected through structured clinical interviews, physical examination, and review of the electronic medical records. Standardized case report forms were used across all participating centres to ensure consistency in data collection. Laboratory tests were performed according to each centre’s routine clinical practice. NT-proBNP assessment was recommended both at admission and within the 48 h preceding hospital discharge, although discharge measurements were not systematically available in all patients. CKD was defined as an estimated glomerular filtration rate (eGFR) < 60 mL/min/1.73 m^2^, calculated using the CKD-EPI equation based on baseline serum creatinine values recorded in the patients’ clinical history.

Transthoracic echocardiography was performed during the index hospitalization, preferably within the first 72 h after admission, by experienced cardiologists with expertise in cardiac imaging. LVEF was quantified using the biplane Simpson method. Additional structural and functional parameters, including left atrial size, valvular abnormalities, and right ventricular function, were assessed according to current international recommendations. Participating centres followed standardized echocardiographic protocols to ensure consistency in image acquisition and interpretation.

Management decisions, including pharmacological therapy, device implantation, and discharge planning, were left to the discretion of the treating cardiology team in accordance with contemporary ESC guidelines and tailored to each patient’s clinical condition. This pragmatic, real-world approach enhances the external validity of the study by reflecting routine clinical practice within a European public healthcare system.

Patients were followed for 12 months after discharge using a comprehensive multimodal strategy that included scheduled outpatient visits, structured telephone interviews conducted by trained personnel, and systematic review of electronic health records. The prespecified primary composite endpoint was all-cause mortality or HF rehospitalization, the latter defined as an unplanned hospital admission lasting at least 24 h due to worsening HF.

The study was conducted in accordance with the ethical principles of the Declaration of Helsinki. All participants provided written informed consent after receiving detailed information about the study objectives and procedures. The study protocol was approved by the Ethics Committee of Hospital Universitario de Burgos (reference CEIM-2547) and subsequently ratified by the institutional review boards of all participating centres. Data were collected and managed in compliance with the European Union General Data Protection Regulation (EU 2016/679) and the Spanish Organic Law 3/2018 on Personal Data Protection and Guarantee of Digital Rights.

Continuous variables are presented as mean ± standard deviation (SD) or median with interquartile range (IQR), as appropriate according to their distribution. Normality was assessed using the Kolmogorov–Smirnov test and visual inspection of Q–Q plots. Categorical variables are presented as frequencies and percentages. Comparisons between groups were performed using the chi-square test or Fisher’s exact test for categorical variables and Student’s *t*-test or Mann–Whitney *U* test for continuous variables, as appropriate.

Potential prognostic factors were evaluated using Cox proportional hazards regression models. Continuous variables were analysed according to their distribution. Given the markedly skewed distribution of NT-proBNP values, this variable was dichotomized at the cohort median (5400 pg/mL) to facilitate interpretation of hazard ratios. Multivariable models were constructed using forward stepwise selection based on likelihood ratios, with a significance level of *p* < 0.05 for variable entry and retention. The proportional hazards assumption was assessed by graphical inspection and statistical testing of Schoenfeld residuals. Model discrimination was evaluated using Harrell’s C-index, whereas calibration was assessed using the Grønnesby–Borgan goodness-of-fit test. Confidence intervals and standard errors were estimated using bootstrap resampling (500 replicates), providing robust non-parametric estimates of statistical precision. All statistical analyses were performed using R version 3.6.1 (R Foundation for Statistical Computing, Vienna, Austria).

Pharmacological variables of interest included guideline-directed therapies with established prognostic benefit in HF: beta-blockers, angiotensin-converting enzyme inhibitors (ACEI), angiotensin receptor blockers (ARB), angiotensin receptor–neprilysin inhibitors (ARNI), mineralocorticoid receptor antagonists (MRA), and sodium–glucose cotransporter 2 inhibitors (SGLT2i). In the subgroup of patients with HFrEF (LVEF ≤40%), Kaplan–Meier survival analysis was performed to evaluate clinical outcomes according to specific combinations of guideline-directed medical therapy (GDMT) prescribed at discharge. These combinations were defined according to the presence or absence of the four evidence-based therapeutic pillars (beta-blockers, ACEI/ARB/ARNI, MRA, and SGLT2i), rather than simply by the total number of drug classes prescribed. Differences between groups were assessed using the log-rank test, and hazard ratios with 95% confidence intervals were calculated.

## 3. Results

Of the 668 patients initially screened, 602 were finally included in the analysis (Figure S1 of the Supplementary Material) after excluding patients who did not meet selection criteria, withdrew consent, were lost to follow-up before completing at least the first month, or died during the index hospitalization. In total, 22 patients died during the index admission and were therefore excluded from the post-discharge prognostic analysis.

Patient recruitment was distributed across participating cardiology departments of different levels of complexity within the Castilla y León healthcare network. These centres differed in size, referral patterns, and local clinical protocols, thereby providing a broad representation of real-world clinical practice in the management of patients hospitalized for AHF (Supplementary Figure S2).

Among the 602 included patients, 37.0% were women, and the mean age was 72.6 ± 12.0 years. Regarding left ventricular systolic function, 47.4% of patients had HFrEF, 12.0% had HF with mildly reduced ejection fraction (HFmrEF), and the remaining patients had HFpEF. The baseline demographic and clinical characteristics of the study population are summarized in Table 1.

**Figure 1:**
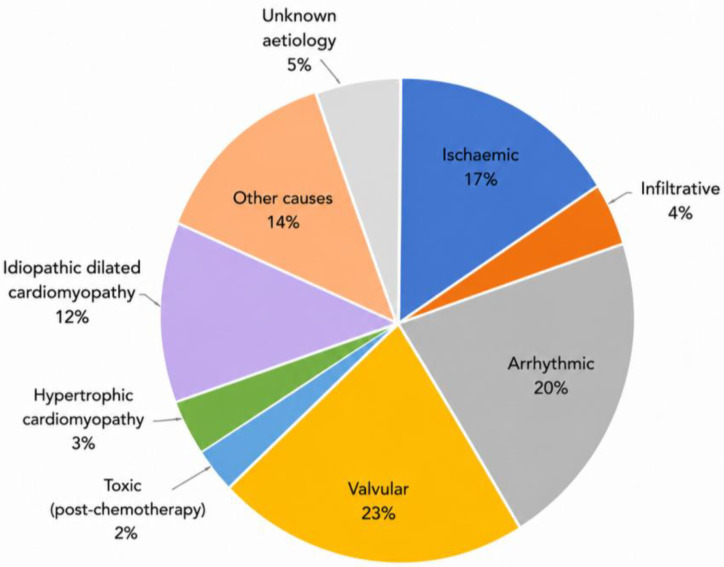
Distribution of the underlying etiologies of heart failure in the RECYLICA cohort (N = 602). Percentages may not total 100% because of rounding. Data on heart failure etiology were unavailable for two patients (0.3%).

Valvular heart disease was the most frequent underlying etiology of AHF (23.3%), followed by arrhythmia-related causes (20.4%) and ischemic heart disease (16.8%). The distribution of the underlying etiologies is shown in Figure 1.

During the index hospitalization, 58 patients (9.6%) required mechanical ventilatory support, 48 (8.0%) received intravenous inotropic therapy, and 15 (2.5%) required temporary mechanical circulatory support because of hemodynamic instability. Intravenous iron therapy was administered to 25.7% of patients, in accordance with contemporary guideline recommendations. Coronary revascularization was performed in 44 patients (7.3%), valve surgery in 14 (2.3%), and coronary artery bypass grafting in 5 (0.8%). Electrical cardioversion was performed in 44 patients (7.3%), mainly to treat atrial arrhythmias contributing to the acute decompensation.

All patients underwent transthoracic echocardiography within the first 72 h of admission. The mean LVEF was 43.0 ± 17.1%. Moderate or greater mitral regurgitation (grade ≥ 2) was observed in 492 patients (81.7%), of whom 43.4% had functional mitral regurgitation. This variable was recorded as an echocardiographic finding during the index hospitalization and should not be interpreted as a formally adjudicated precipitating cause of AHF.

The mean length of hospital stay was 9 ± 7 days. At discharge, 64.0% of patients were referred to general cardiology outpatient clinics, whereas 30.2% were followed in specialized HF units. The remaining patients continued follow-up in Internal Medicine or Primary Care. Laboratory parameters at discharge showed a mean hemoglobin concentration of 12.93 ± 2.35 g/dL and a mean eGFR of 59.2 ± 24.8 mL/min/1.73 m^2^. Median NT-proBNP levels decreased from 4763 pg/mL (IQR 2557–9923) at admission to 1888 pg/mL (IQR 949–4285) at discharge, consistent with clinical decongestion. NT-proBNP values were unavailable in 199 patients at discharge and in 32 patients at admission.

Pharmacological treatment at discharge is summarized in Table 2 according to LVEF phenotype. Patients with HFrEF were more frequently prescribed ARNI, MRA, beta-blockers, and SGLT2i than those with HFpEF. In contrast, ACEI/ARB therapy was more common among patients with HFpEF, whereas loop diuretics were widely prescribed in both groups, with slightly higher use in patients with HFpEF.

During the 12-month follow-up, 83 patients (13.8%) died, including 32 (5.3%) from cardiovascular causes. In addition, 105 patients (17.4%) were rehospitalized because of AHF. Overall, the primary composite endpoint of all-cause mortality or HF rehospitalization occurred in 158 patients (26.2%).

Because NT-proBNP values showed a markedly skewed distribution, this variable was dichotomized at the cohort median (5400 pg/mL) to facilitate the clinical interpretation of hazard ratios in the regression models. Multivariable Cox regression analysis identified admission NT-proBNP > 5400 pg/mL, previous HF, AF or atrial flutter, CKD, COPD, previous ICD implantation, and higher LVEF as independent predictors of the composite endpoint (Table 3). The final model demonstrated good discrimination (Harrell’s C-index = 0.708) and adequate calibration according to the Grønnesby–Borgan goodness-of-fit test (*p* = 0.124). To further support model performance, the Schoenfeld residual plots and calibration plot have been included in the revised Supplementary Material (Figures S3 and S4).

In the overall multivariable model for the composite endpoint, none of the individual discharge pharmacological therapies remained independently associated with outcome, so we performed a focused survival analysis in the subgroup of patients with HFrEF (EF ≤40%) to evaluate the impact of the completeness of neurohormonal therapy.

**Figure 2:**
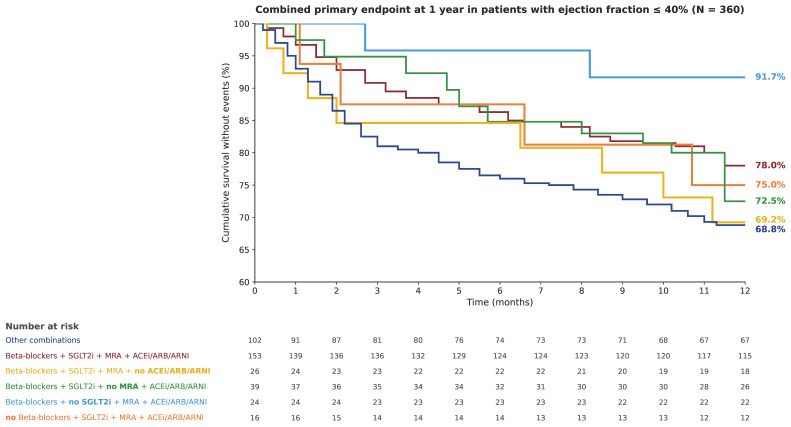
Kaplan–Meier curves for the composite endpoint of all-cause mortality or heart failure rehospitalization according to specific guideline-directed medical therapy (GDMT) combinations at hospital discharge in patients with heart failure with reduced left ventricular ejection fraction (HFrEF; LVEF ≤ 40%). The number of patients at risk is displayed below the graph. ACEI, angiotensin-converting enzyme inhibitor; ARB, angiotensin II receptor blocker; ARNI, angiotensin receptor–neprilysin inhibitor; MRA, mineralocorticoid receptor antagonist; SGLT2i, sodium–glucose cotransporter 2 inhibitor.

For the HFrEF subgroup, GDMT completeness was defined as discharge treatment with four therapeutic pillars: a beta-blocker, an SGLT2 inhibitor, an MRA, and a RAAS blocker (ACEI/ARB or ARNI). Kaplan–Meier analysis was performed according to specific combinations of GDMT classes at discharge. Patients discharged with incomplete combinations had significantly worse outcomes than those receiving the more complete combinations shown in Figure 2.

A secondary multivariable analysis focused on all-cause mortality alone (Table S1 in the Supplementary Material) identified advanced age, elevated admission NT-proBNP, prior AF/flutter, prior ICD, chronic kidney disease, and beta-blocker therapy at discharge as independent predictors of death during follow-up. In contrast, treatment with ACEI/ARB/ARNI at discharge was independently associated with lower mortality, consistent with the established prognostic benefit of renin–angiotensin system inhibition in patients with HF.

## 4. Discussion

Our real-world, regional, population-based registry provides contemporary insights into the clinical characteristics, management, and outcomes of patients hospitalized with AHF. Our findings indicate that the clinical course following hospitalization for acute decompensation is primarily determined by three major factors: the burden of comorbidities (particularly AF, CKD, and COPD), elevated admission NT-proBNP levels, and a history of HF, reflected by both a previous diagnosis and prior ICD implantation. Of particular clinical interest, patients with higher LVEF appeared to experience worse outcomes. However, this finding should be interpreted with caution, as it was not confirmed in the mortality-only model, suggesting that the observed association is driven predominantly by HF rehospitalization rather than mortality.

These findings are particularly relevant given the limited availability of contemporary real-world data on AHF management from cardiology departments within our healthcare system. Although a national registry of patients with HF managed in hospitals and specialized HF units is currently underway, only preliminary baseline data have been published to date. Among the few Spanish registries reporting clinical outcomes, one included more than 5000 patients presenting to emergency departments with HF, of whom 76.1% required hospitalization [9], whereas another focused exclusively on patients admitted to Internal Medicine departments [10]. Both registries mainly included patients treated before 2011, with only one extending recruitment until 2016 and reporting outcomes in 3550 patients hospitalized with AHF in Internal Medicine departments [11]. Since then, substantial advances have occurred in both diagnostic assessment and HF treatment. Consequently, our registry provides more contemporary evidence on the characteristics, management, and prognostic factors of patients admitted to cardiology departments and treated according to current pharmacological and non-pharmacological standards of care.

The baseline clinical and demographic characteristics of our cohort were broadly comparable to those reported in the European Society of Cardiology Heart Failure Long-Term Registry (ESC-HF-LT-R), including similar proportions of women and comparable prevalences of AF, diabetes mellitus, hypertension, CKD, and HFpEF [4]. However, the proportion of patients with de novo HF was considerably higher in our registry (58.1% vs. 29.1% in the ESC-HF-LT-R) [4]. This difference may reflect variations in referral pathways, healthcare utilization, patient selection, or regional epidemiology and deserves further investigation. In addition, the underlying etiology of HF differed substantially between the two registries. Whereas ischemic heart disease represented the leading etiology in the ESC-HF-LT-R (approximately 50%, depending on the participating region), it accounted for only 16% of cases in our cohort, while valvular heart disease was the predominant etiology. This discrepancy is most likely explained by the inclusion of patients with acute coronary syndromes in the ESC-HF-LT-R, who were specifically excluded from our study. Our findings are also consistent with reports from other developed countries, including Japan [12], whereas compared with non-European registries, our cohort included older patients with a higher prevalence of cardiovascular risk factors, AF, and CKD [13,14].

The pharmacological treatment patterns observed in our study provide valuable insight into real-world implementation of GDMT. As expected, patients with HFrEF were more frequently prescribed ARNI, MRA, beta-blockers, and SGLT2i, whereas ACEI/ARB therapy was prescribed more often in patients with HFpEF (Table 2). Overall, the prescription rates of guideline-recommended therapies were high for a population hospitalized with AHF, particularly considering the large proportion of patients with de novo HF. These findings are consistent with those reported in the ESC-HF-LT-R [6], in which the prescription rates of ACEI/ARB/ARNI, MRA, beta-blockers, and SGLT2i were 74.7%, 68.15%, 83.24%, and 72.2%, respectively. Nevertheless, despite robust evidence supporting the use of SGLT2i in patients with HFpEF [15,16], their prescription remained significantly lower in this subgroup, highlighting an important opportunity to further optimize evidence-based therapy. Conversely, loop diuretics were prescribed more frequently in patients with HFpEF, probably reflecting the continued reliance on symptom-oriented treatment in this population.

Our multivariable analysis identified several independent predictors of the composite endpoint of all-cause mortality or HF rehospitalization, in agreement with previous real-world studies. AF [17] was strongly associated with adverse outcomes and probably reflects the complex interaction between rhythm disturbances, hemodynamic impairment, and neurohormonal activation. Likewise, COPD [18], previous HF, elevated NT-proBNP levels, and CKD [6,13,14] were independently associated with a worse prognosis, underscoring the growing impact of multimorbidity on outcomes in patients with AHF. In contrast, although diabetes mellitus was highly prevalent in our cohort, it was not independently associated with the primary endpoint, differing from several previous registries in which diabetes has been associated with increased mortality and rehospitalization [19].

The association between prior ICD implantation and poorer outcomes should not be interpreted as a harmful effect of the device itself. Rather, ICD carriers in this cohort likely represent a subgroup with more advanced structural heart disease, greater arrhythmic burden, more severe left ventricular dysfunction, and a higher baseline risk profile. This interpretation is supported by the fact that prior ICD was associated not only with the composite endpoint but also with all-cause mortality in the mortality-specific model. Therefore, ICD presence should be regarded as a marker of disease severity and residual confounding by indication cannot be excluded in this observational registry.

NT-proBNP remains one of the most robust biomarkers for risk stratification in HF, both in acute and chronic settings [20]. Although previous studies have shown that changes in NT-proBNP during hospitalization provide important prognostic information [21], our study demonstrates that admission NT-proBNP alone independently predicts adverse outcomes in a contemporary real-world cohort of patients hospitalized with AHF. This finding highlights the value of early biomarker assessment for identifying high-risk patients and supports its incorporation into routine clinical risk stratification to guide treatment decisions and discharge planning.

A previous history of HF was also independently associated with worse clinical outcomes. Our findings are consistent with those from the Egyptian ESC-HF-LT-R cohort, in which patients admitted with acute decompensation of chronic HF had higher one-year mortality than those with de novo HF [22]. Similar observations have been reported in a Danish nationwide registry, where acute exacerbations of chronic HF were associated with poorer survival than de novo presentations [23], probably reflecting more advanced structural heart disease and irreversible myocardial remodeling. Likewise, the Spanish Registro de Insuficiencia Cardiaca Aguda(RICA) reported lower all-cause mortality among patients with de novo AHF than among those with acute decompensation of chronic HF [11]. In addition, greater comorbidity burden and renal dysfunction were identified as independent predictors of mortality in the RICA registry. Collectively, these findings support the concept that a previous diagnosis of HF is a marker of cumulative disease burden and more advanced disease.

In our cohort, higher LVEF was independently associated with an increased risk of the composite endpoint. However, this finding should be interpreted with caution, as the association was not observed in the mortality-only model, suggesting that it is primarily driven by HF rehospitalization rather than mortality. Previous U.S. registries have reported similar outcomes among patients with preserved and reduced LVEF [24,25], whereas the ESC-HF-LT-R found higher rates of cardiovascular mortality and HF readmission among patients with HFrEF, while patients with HFpEF experienced a greater incidence of non-cardiovascular events [6]. Similarly, the RICA registry reported more cardiovascular deaths among patients with HFrEF and a higher proportion of non-cardiovascular deaths among those with HFpEF or HFmrEF [26]. In our study, patients with higher LVEF were generally older and had a greater prevalence of AF and other comorbidities, which may partly explain these findings.

When analyzed individually, none of the four foundational therapies for HFrEF remained independently associated with the composite endpoint in the multivariable analysis. Although these treatments have well-established prognostic benefits in randomized clinical trials, observational registries are inherently susceptible to confounding by indication, and the inclusion of a substantial proportion of patients with HFpEF may have further reduced the ability to detect independent treatment effects. In the secondary multivariable analysis focused on all-cause mortality, however, discharge treatment with ACEI/ARB/ARNI was independently associated with lower mortality, whereas beta-blocker therapy was associated with higher mortality. These findings should be interpreted cautiously, as they most likely reflect differences in baseline disease severity and treatment indication rather than causal effects of the therapies themselves. Because no propensity-adjusted or treatment-weighted analysis was performed, residual confounding by indication cannot be excluded.

Importantly, our findings provide additional real-world evidence regarding the prognostic implications of incomplete implementation of GDMT at hospital discharge. Among patients with HFrEF, those discharged receiving less comprehensive GDMT combinations experienced significantly higher rates of the primary composite endpoint than those treated with more complete combinations incorporating the four foundational therapeutic classes. These findings are consistent with previous studies highlighting the prognostic importance of optimizing pharmacological therapy before hospital discharge [27,28,29,30] and further support the feasibility and safety of early initiation of multiple evidence-based therapies [31,32,33]. Moreover, the STRONG-HF trial demonstrated that rapid optimization of guideline-directed therapy during hospitalization, followed by intensive post-discharge follow-up, improves clinical outcomes [34]. Taken together, these findings support the early initiation of all four foundational GDMT classes during hospitalization whenever clinically feasible and reinforce the concept that achieving greater therapeutic completeness may be more important than maximizing the dose of a limited number of agents.

The markedly higher proportion of de novo HF observed in RECYLICA compared with ESC-HF-LT-R may be related to differences in case mix and patient selection. RECYLICA specifically included patients admitted to cardiology departments and excluded acute coronary syndromes, which may have selected a different clinical profile from broader all-comer AHF registries. In addition, regional referral pathways and local admission practices may have influenced the proportion of first-presentation HF. These differences should be considered when comparing event rates and the external validity of prognostic models across registries.

Beyond their pathophysiological relevance, the predictors identified in this registry may also be useful as building blocks for pragmatic risk stratification tools in routine clinical practice. Variables such as age, renal dysfunction, previous HF, atrial arrhythmias, natriuretic peptide burden, and treatment profile are readily available during hospitalization and could help identify patients at higher risk of post-discharge events who may benefit from closer follow-up and more intensive therapeutic optimization.

Despite the strengths of our study, several limitations should be acknowledged. First, the observational design precludes causal inference and remains susceptible to residual confounding despite multivariable adjustment. In particular, treatment-related associations should be interpreted cautiously because no propensity score or other causal inference analyses were performed.

Second, the registry included only patients admitted to Cardiology departments. Although participating centres represented different levels of complexity within the Castilla y León healthcare network, with heterogeneous referral patterns and local management protocols, patients admitted to Internal Medicine, Nephrology, or other specialties were not included. Consequently, frailer or more comorbid patients may have been underrepresented, potentially limiting the generalizability of our findings to the overall AHF population. Conversely, this design allowed the inclusion of patients in whom AHF was the primary reason for admission, minimizing the influence of competing acute conditions. This approach may be clinically relevant, as previous propensity-matched analyses have suggested that the admitting specialty may influence prognosis in patients hospitalized for AHF [35].

Third, to improve cohort homogeneity, we excluded patients with AHF secondary to acute coronary syndromes or bradyarrhythmias. Although these conditions represent important causes of admission to Cardiology departments, they constitute distinct clinical entities with different pathophysiological mechanisms and therapeutic strategies. Their exclusion may therefore limit the applicability of our findings to the entire spectrum of patients presenting with AHF.

Fourth, patients who died during the index hospitalization were not included in the post-discharge prognostic analysis. Consequently, the sickest patients with the highest early mortality risk were excluded, introducing a potential survivorship bias and preventing assessment of predictors of in-hospital mortality.

Fifth, NT-proBNP was systematically collected at admission but was unavailable at discharge in 199 patients. Therefore, we were unable to evaluate the prognostic significance of changes in NT-proBNP during hospitalization. Likewise, recently proposed laboratory biomarkers with potential prognostic value in AHF, including electrolyte-related biomarkers and other emerging biochemical markers, were not systematically collected and therefore could not be evaluated.

Finally, we did not perform analyses stratified according to HF etiology or by participating centre. Although patient recruitment was distributed across hospitals with different organizational characteristics and clinical practice patterns, centre-specific effects cannot be completely excluded.

## 5. Conclusions

The RECYLICA registry provides contemporary real-world data on the clinical characteristics, management, and prognosis of patients hospitalized with AHF in cardiology departments. Adverse outcomes were primarily associated with a greater burden of comorbidities, elevated admission NT-proBNP levels, and a previous history of HF. In addition, patients with higher LVEF experienced a higher risk of the composite endpoint, an association that appeared to be driven mainly by HF rehospitalization.

Among patients with HFrEF, less comprehensive implementation of GDMT at discharge was associated with significantly worse outcomes, reinforcing the importance of early initiation of all four foundational therapies whenever clinically feasible. These findings support the implementation of structured in-hospital optimization strategies and contribute contemporary real-world evidence to improve risk stratification and management of patients hospitalized with AHF.

## Figures and Tables

**Table 1:** Baseline characteristics of the sample.

Characteristics	N (602)
Women	223 (37%)
Age (years)	72.6 ± 12.0
High blood pressure	408 (67.9%)
Diabetes mellitus	226 (37.5%)
Active smoker or ex-smoker	273 (47.2%)
COPD	82 (13.7%)
Chronic kidney failure	185 (30.8%)
Ictus	62 (10.3%)
Coronary artery disease	105 (17.6%)
Charlson scale (points)	4.0 ± 2.3
De novo AHF	350 (58.1%)
AF or atrial flutter	251 (41.7%)
Previous ICD	27 (4.5%)
Previous CRT	11 (1.8%)
Baseline NYHA class	
I	233 (45.9%)
II	219 (43.1%)
III	53 (10.4%)
IV	3 (0.6%)
LVEF on admission (%)	43.08 ± 17.10
Hemoglobin on admission (g/dL)	13.08 ± 2.26
Glomerular filtration rate on admission (ml/min/1.73 m^2^)	60.86 ± 25.90
Ferritin on admission (ng/mL)	141.5 [63.0–260.0]
Transferrin saturation index on admission (%)	18.68 ± 12.25
NT-proBNP on admission (pg/mL)	4763 [2257–9923]
High-sensitivity troponin T on admission (ng/L)	36.0 [19.5–69.1]

ICD: implantable cardioverter-defibrillator; COPD: chronic obstructive pulmonary disease; AF: atrial fibrillation; AHF: acute heart failure; NT-proBNP: N-terminal pro-brain natriuretic peptide type B; CRT: cardiac resynchronization therapy.

**Table 2:** Treatment prescribed at discharge based on the type of heart failure.

Pharmacological Group	HF with EF ≤ 40% (N: 360)	HF with EF > 40% (N: 240)	*p*
ACEI/ARB	102 (28.50%)	118 (49.20%)	<0.001
ARNI	166 (46.2%)	5 (2.1%)	<0.001
MRA	244 (68.0%)	96 (40.2%)	<0.001
SGLT2i	260 (72.2%)	108 (45.2%)	<0.001
Beta blockers	298 (83.0%)	148 (61.7%)	<0.001
Loop diuretic	300 (83.30%)	215 (88.6%)	0.031

ARB: angiotensin-2 receptor antagonist; MRA: mineralocorticoid receptor antagonist; ARNI: angiotensin receptor neprilysin inhibitor; EF: left ventricular ejection fraction; HF: heart failure; ACEI: angiotensin-converting enzyme inhibitor; SGLT2i: sodium-glucose cotransporter 2 inhibitor. Two patients had missing EF classification and were therefore excluded from the EF-stratified treatment table, which is why Table 2 totals 600 rather than 602.

**Table 3:** Independent predictors of the composite event of death or admission for heart failure at one year.

Variable	Hazard Ratio	95% Confidence Interval	*p*
NT-proBNP on admission > 5400 pg/mL	1626	1138–2323	0.008
Previous Heart Failure	1811	1245–2636	0.002
Ejection Fraction according to SIMPSON (%)	1012	1002–1023	0.021
Previous Atrial Fibrillation/Flutter	1596	1103–2310	0.013
Previous Implantable Cardioverter Defibrillator	2835	1587–5066	<0.001
Chronic Kidney Disease	1592	1100–2304	0.014
Chronic Obstructive Pulmonary Disease	1607	1058–2441	0.026
Harrell’sc: 0.708 Jackknife 95% Confidence Interval [0.668–0.748], *p* < 0.001; Groennesby and Borgan test *p* = 0.124			

Variables included in the model: age, diabetes, sex, Charlson index, NT-proBNP at admission, previous HF, ejection fraction, implantable cardioverter-defibrillator, chronic obstructive pulmonary disease, chronic kidney disease, previous atrial fibrillation/flutter, significant valvular disease, coronary artery disease, beta-blocker intake, aldosterone antagonist intake, renin–angiotensin–aldosterone system inhibitor intake, SGLT2i intake, digoxin intake, diuretic intakeNT-proBNP: N-terminal pro-brain natriuretic peptide type B.

## Data Availability

The data presented in this study are available on request from the corresponding author due to ethical restrictions.

## Supplementary Materials

The following supporting information can be downloaded at: https://www.mdpi.com/article/10.3390/jcdd13070310/s1; Figure S1. Flowchart of selected patients; Figure S2. Distribution of patients by hospital; Figure S3. Proportional hazards verification based on Schoenfeld residuals; Figure S4. Calibration chart by decile and survival stratified by risk quintiles; Table S1. Analysis of predictors of mortality during follow-up.

## Abbreviations

ACEI	Angiotensin-Converting Enzyme Inhibitor
AF	Atrial Fibrillation
AHF	Acute Heart Failure
ARB	Angiotensin Receptor Blocker
ARNI	Angiotensin Receptor–Neprilysin Inhibitor
CKD	Chronic Kidney Disease
COPD	Chronic Obstructive Pulmonary Disease
EF	Ejection Fraction
ESC	European Society of Cardiology
ESC-HF-LT-R	European Society of Cardiology Heart Failure Long-Term Registry
GDMT	Guideline-Directed Medical Therapy
HFrEF	Heart Failure with Reduced Ejection Fraction
HFmrEF	Heart Failure with Mildly Reduced Ejection Fraction
HFpEF	Heart Failure with Preserved Ejection Fraction
ICD	Implantable Cardioverter-Defibrillator
IQR	Interquartile Range
LVEF	Left Ventricular Ejection Fraction
MRA	Mineralocorticoid Receptor Antagonist
NT-proBNP	N-terminal pro-B-type Natriuretic Peptide
RAASi	Renin–Angiotensin–Aldosterone System Inhibitor
RICA	Registro de Insuficiencia Cardíaca Aguda
ROC	Receiver Operating Characteristic
SD	Standard Deviation
SGLT2i	Sodium–Glucose Co-Transporter 2 Inhibitor
STRONG-HF	Safety, Tolerability and Efficacy of Rapid Optimization, Helped by NT-proBNP Testing, of Heart Failure Therapies
