# Supplementary material for: Prognostic Factors of Acute Heart Failure: A Regional Population Registry
Source: J Cardiovasc Dev Dis. 2026 Jul 6;13(7):310. doi: 10.3390/jcdd13070310 (PMC13409770; doi:10.3390/jcdd13070310)
Supplement: Supplementary file 1 [file jcdd-13-00310-s001.zip › jcdd-4338503-supplementary.pdf]

SUPPLEMENTARY MATERIAL

Figure S1. Flowchart of selected patients.

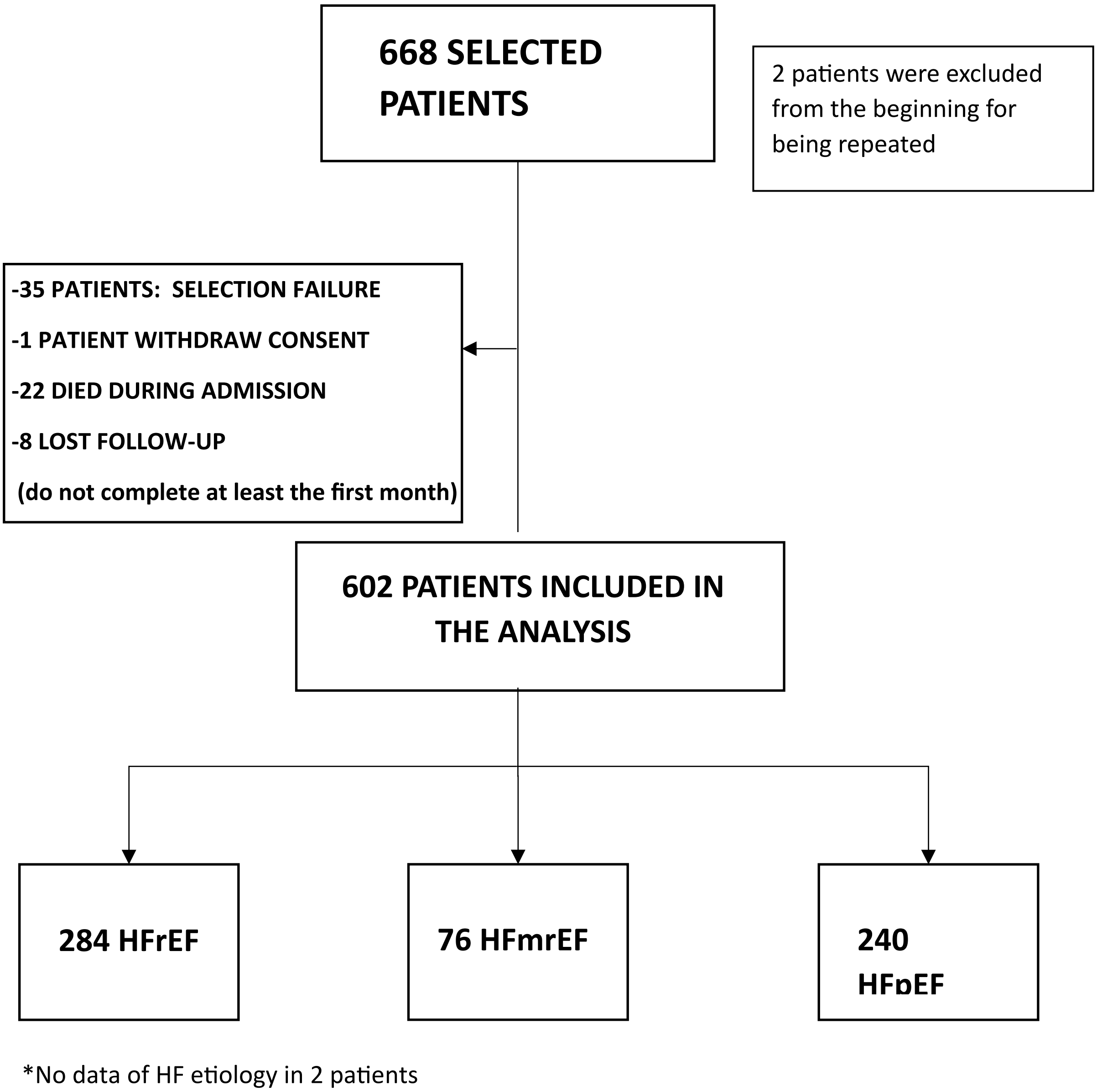

HF: heart failure; HFmrEF: heart failure with midly reduced ejection fraction; HFpEF: heart failure with preserved ejection fraction; HFrEF: heart failure with reduced ejection fraction

Figure S2. Distribution of patients by hospital

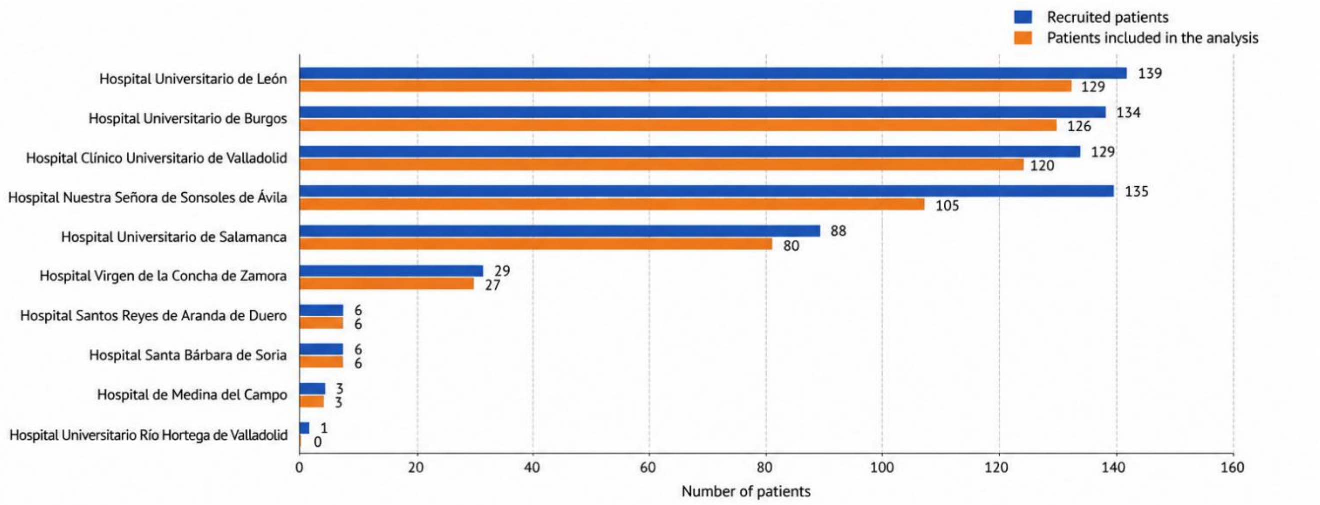

No.	Hospital	Recruited patients	Patients included in the analysis
1	Hospital Universitario de León	139	129
2	Hospital Universitario de Burgos	134	126
3	Hospital Clínico Universitario de Valladolid	129	120
4	Hospital Nuestra Señora de Sonsoles de Ávila	135	105
5	Hospital Universitario de Salamanca	88	80
6	Hospital Virgen de la Concha de Zamora	29	27
7	Hospital Santos Reyes de Aranda de Duero	6	6
8	Hospital Santa Bárbara de Soria	6	6
9	Hospital de Medina del Campo	3	3
10	Hospital Universitario Río Hortega de Valladolid	1	0

Supplementary Figure S2. Distribution of recruited and included patients across participating hospitals. Hospitals are ordered according to the number of patients included in the final analysis.

Figure S3. Proportional hazards verification based on Schoenfeld residuals

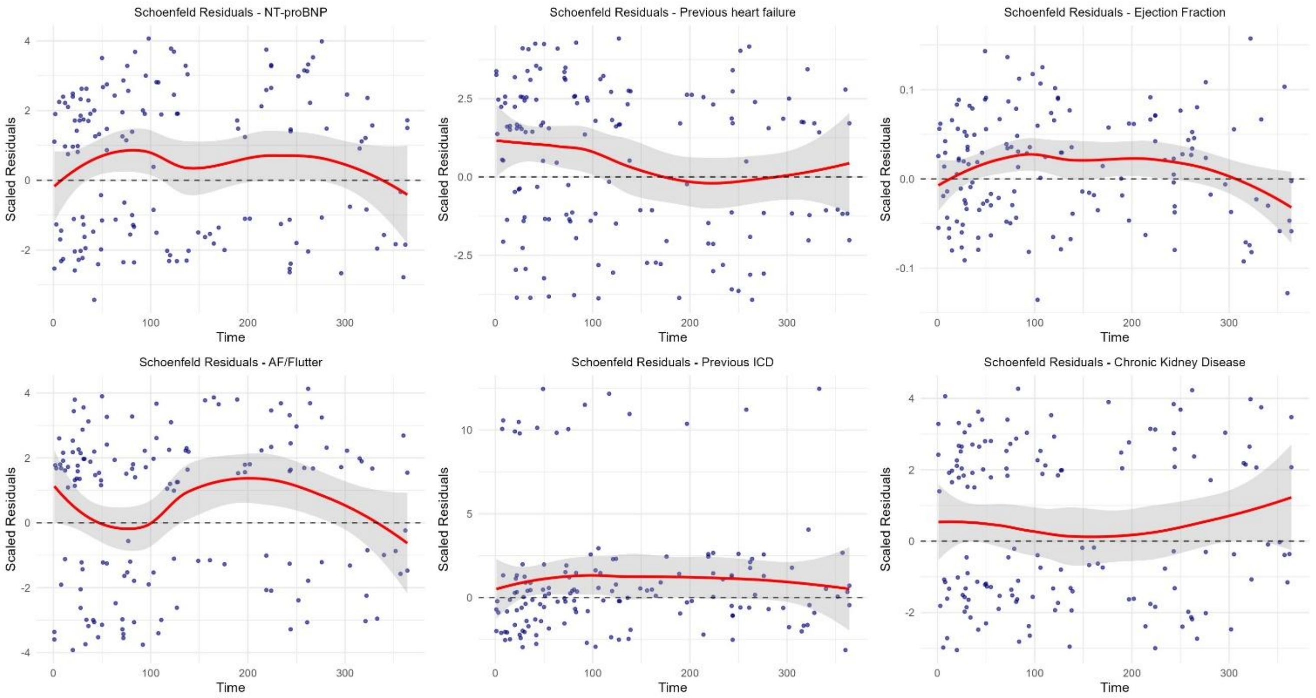

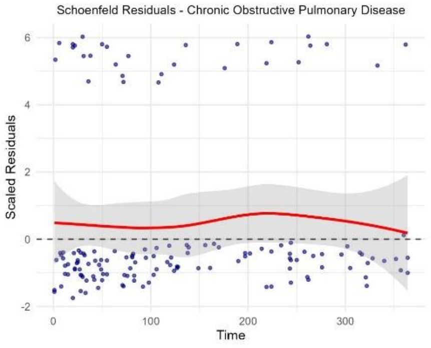

Schoenfeld Residuals Test for Proportional Hazards

	Rho	Chi2	df	Prob>chi2
NT-proBNP	-0.00101	0.00	1	0.9908
Previous heart failure	-0.16746	2.21	1	0.1369
Ejection Fraction	-0.04985	0.31	1	0.5778
AF/Flutter	0.02563	0.10	1	0.7520
Previous ICD	0.00065	0.00	1	0.9936
Chronic Kidney Disease	0.03727	0.19	1	0.6668
Chronic Obstructive Pulmonary Disease	0.01356	0.03	1	0.8733
Global test		4.84	7	0.6789

Figure S4. Calibration chart by decile and survival stratified by risk quintiles.

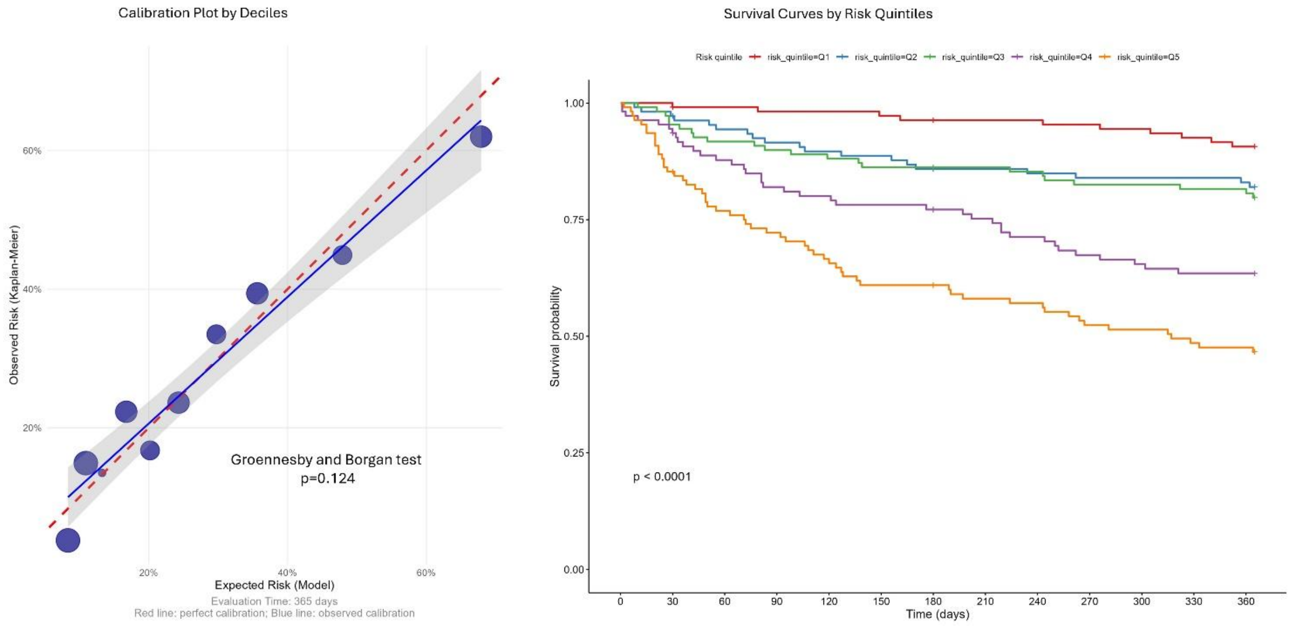

Table S1. Analysis of predictors of mortality during follow-up

Variable	Univariate Analysis			Multivariate Analysis		
	Hazard Ratio	95% Bootstrap Confidence Interval	P-value	Hazard Ratio	95% Bootstrap Confidence Interval	P-value
Age	1.053	1.032 – 1.074	<0.001	1.053	1.024 –1.083	<0.001
Sex	1.333	0.873 – 2.034	0.183			
Diabetes	1.045	0.664 – 1.645	0.850			
Charlson index >3	3.469	1.759 – 6.842	<0.001			
NT-probnp > 5400 pg/ml	2.857	1.795 – 4.549	<0.001	2.785	1.740 – 4.457	<0.001
Previous heart failure	2.557	1.601 – 4.081	<0.001			
LVEF (%)	1.004	0.992 – 1.017	0.510			
AF/Previous Atrial Flutter	3.551	2.243 – 5.623	<0.001	2.208	1.345 – 3.625	0.002
Moderate or severe valve disease	2.667	1.783 – 3.987	<0.001			
Previous DAI	3.913	1977 – 7743	<0.001	5.237	2.438 – 11.247	<0.001
Coronary Artery Disease	1.546	0.931 – 2.569	0.092			
Chronic Kidney Failure	2.415	1.557 – 3.746	<0.001			
COPD	1.419	0.786 – 2.562	0.245			
Beta blockers	1.467	0.825 – 2.611	0.192	2.211	1.186– 4.122	0.013
ARM	1.108	0.710 – 1.727	0.652			
iSLGT2	0.751	0.486 – 1.159	0.195			
iSRAA	0.431	0.277 – 0.669	<0.001	0.483	0.300 – 0.778	0.003
Digoxin	1.058	0.485 – 2.308	0.887			
Loop diuretic	1.390	0.644 – 2.996	0.401			
Harrell'sc: 0.774 Jackknife 95% CI [0.722 – 0.827]. p<0.001; Groennesby and Borgan test p=0.353						

MRAs: mineralocorticoid receptor antagonists; ICDs: implantable cardioverter-defibrillators; COPD: chronic obstructive pulmonary disease; AF: atrial fibrillation; LVEF: left ventricular ejection fraction; SGLT2i: sodium-glucose cotransporter inhibitors; RAASi: renin–angiotensin–aldosterone system inhibitors
